# Effect of ultra-low-dose estriol and lactobacilli vaginal tablets (Gynoflor®) on inflammatory and infectious markers of the vaginal ecosystem in postmenopausal women with breast cancer on aromatase inhibitors

**DOI:** 10.1007/s10096-015-2447-1

**Published:** 2015-07-30

**Authors:** G. Donders, G. Bellen, P. Neven, P. Grob, V. Prasauskas, S. Buchholz, O. Ortmann

**Affiliations:** Department of Obstetrics and Gynecology, University Antwerp, Antwerp, Belgium; Femicare vzw, Clinical Research for Women, Gasthuismolenstraat 31, 3300 Tienen, Belgium; Department of Obstetrics and Gynecology, Gasthuisberg Hospital, University Leuven, Leuven, Belgium; Department of Obstetrics and Gynecology, University Medical Center Regensburg, Regensburg, Germany; Medinova AG, Zurich, Switzerland

## Abstract

This study was a detailed microscopic analysis of the changes of vaginal microflora characteristics after application of 0.03 mg estriol-lactobacilli combination on the vaginal ecosystem in postmenopausal breast cancer (BC) survivors on aromatase inhibitors (AI) with severe atrophic vaginitis. A total of 16 BC women on AI applied daily one vaginal tablet of Gynoflor® for 28 days followed by a maintenance therapy of three tablets weekly for 8 weeks. During four follow up visits a smear from the upper lateral vaginal wall was analysed by phase contrast microscopy at 400 times magnification in order to classify the lactobacillary grades(LBG), bacterial vaginosis (BV), aerobic vaginitis (AV), vulvovaginal candidosis (VVC), proportional number of leukocytes and evidence of parabasal cells and epitheliolysis. LBG improved from 81 % LBG-III at entry to 88 % LBG-I&IIa after 2 weeks of initial therapy, which further improved upon follow up (*p* < 0.001). Whereas BV was a rare event, AV was frequent and substantially improved during treatment (*p* < 0.01). While at entry most patients had moderate or severe AV, after maintenance therapy no patient except one had AV. The number of leukocytes dropped dramatically from a score of 1.78 ± 0.70 to 1.06 ± 0.25 which was consistent till the end of the study (*p* < 0.01). Parabasal cells dropped from a score of 3.4 ± 0.64 at entry to 1.3 ± 0.60 at the final visit (*p*_trend_ < 0.01). Starting from a low rate of *Candida* colonisation of 2/14 (14 %), a sudden rise to 7/16 (44 %) occurred after 2 weeks, to return back to base levels at susequent visits. The vaginal use of ultra-low dose estriol and lactobacilli results in rapid and enduring improvement of all markers of the vaginal microflora and epithelial vaginal cell quality in women with breast cancer on AI with dyspareunia. *Candida* may develop soon after its use, but rapidly disappears again upon their prolonged use. Due to its excellent safety profiles and clinical efficacy we recommend this product as first choice in women on AI with severe dyspareunia.

## Introduction

A healthy vaginal ecosystem depends on a normal flora mainly consisting of Lactobacillus spp., a sufficient estrogen-dependent maturation of the vaginal epithelium and an intact local immunity [1–4]. Estrogen deprivation therapy installed in hormone receptor positive breast cancer women prolongs survival [5], but invariably leads to symptomatic vaginal atrophy or even aerobic vaginitis in both pre- and post-menopausal women. The most common symptoms of vaginal atrophy include dryness, dyspareunia, vaginal burning, discharge and pain. These symptoms are paralleled by cytological changes which, in a vaginal smear, manifest as a shift from superficial cell towards parabasal and intermediate cells [6]. Atrophy of vulva, introitus and vagina can be especially problematic for women who want to stay sexually active but experience pain during sexual intercourse due to dryness and atrophic changes [7–9], affecting sexual quality [10].

Epithelial atrophy of the vagina can be treated by the oral or vaginal administration of estriol, but according to the Consensus Statement on Menopausal Hormone Therapy, local therapy with low-dose estrogen is preferred for women whose symptoms are limited to vaginal dryness or associated discomfort with intercourse [11]. However, estrogen is readily absorbed through the vaginal wall [10], leading to similar systemic effects as would be produced by oral therapy with a 10–20 fold greater dose [12]. Fortunately, only very small dosages are needed to treat vaginal symptoms, and low-potency estrogens like estriol (E3) can readily be used with only limited systemic effects in spite of absorption [10].

Gynoflor is a well-established medicinal product containing 100 million viable Lactobacillus acidophilus KS400 and a ultra low dose of 30 μg E3. It has been introduced to promote the proliferation and maturation of the vaginal epithelium and to enhance the restoration of the vaginal ecosystem [13–16].

In a double-blind, placebo-controlled study Jaisamrarn et al. [17] demonstrated excellent short-term and long-term efficacy of Gynoflor® in menopausal women with symptomatic atrophic vaginitis. In an ambitious pharmacokinetic (PK) study we assessed circulating systemic estrogens in breast cancer (BC) patients on a non-steroidal aromatase inhibitor (NSAI) after insertion of Gynoflor® in vagina affected by severe atrophy [5]. Compared with baseline, serum estrone (E1) and estradiol (E2) did not increase in any of the women at any time following vaginal application. Serum E3 transiently increased after the first application in 15 of 16 women, with a maximum of 168 pg/ml 2–3 hours post-insertion. After 4 weeks, serum E3 was only slightly increased in eight of 16 women with a maximum of 44 pg/ml, without causing any relevant change in serum LH (luteinising hormone), FSH (follicle stimulating hormone) and SHBG (sex hormone binding globulin) concentrations. It was concluded that local vaginal therapy with ultra-low-dose 0.03 mg estriol-lactobacilli combination (Gynoflor®) in postmenopausal BC survivors on aromatase inhibitors (AIs) reporting atrophic vaginitis is a pharmacologically safe treatment with a positive impact on quality of life and sexual activity [5].

In this contribution we analyse the changes of vaginal microflora characteristics in detail in an effort to explain the excellent efficacy of ultra-low-dose 0.03 mg estriol-lactobacilli combination (Gynoflor®) on the vaginal ecosystem in these women and normalisation in postmenopausal BC survivors on AIs with severe atrophic vaginitis.

## Subjects and methods

The safety data and main results of this Phase I pharmacokinetic (PK) study (EudraCT No: 2010-022007-22) have been reported earlier [5]. Sixteen women on NSAI with severe vaginal atrophy (Entry visit, E) applied daily one vaginal tablet of Gynoflor® for 28 days followed by a maintenance therapy of three tablets weekly for 8 weeks and were seen at four follow up visits (C1-C4), where a smear was taken from the upper lateral vaginal wall after introduction of a water lubricated, small size speculum. pH was measured and the smear was numbered and air-dried. Before sending the smears to the central lab (Femicare, Tienen, Belgium), the slides were renumbered according to a computer-generated random table, in order to prevent possible recognition of the microscopist of the nature or order of the visit on which the slide was taken. This code was broken only after complete finalization of the patient follow up data and after the database was locked.

**Fig. 1:**
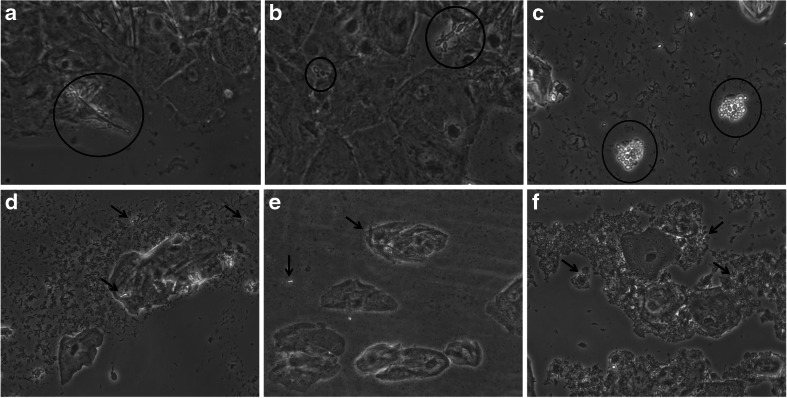
Rehydrated air-dried microscopy pictures of *Candida* (**a**-**c**) and possible *Candida* (**d**-**f**). **a** *Candida* pseudo-mycelium is visualized (*circle*) in a background of lactobacillary grade (LBG) IIB, normal superficial cells, and no inflammation. **b** Beginning Candida pseudo-mycelium (*large circle*) and blastospores (*small circle*) are visualized in a background of LBG IIB, normal superficial cells, and no inflammation. **c** Multiple blastospores (*both circles*) suggestive of *non-albicans Candida* in a background of LBG IIA, normal superficial cells, and no inflammation. In pictures **d**, **e** and **f** structures are encountered that somewhat resemble *Candida* organelles, but insufficiently pathognomonic to ascertain a diagnosis. In such cases extra cultures or PCR may be required to confirm the diagnosis. In (**d**) the background is LBG 1, in (**e**) LBG III, and in (**f**) LBG I, small lactobacillary morphotypes. In all pictures maturation index is high and there is no inflammation

Microscopy was done in a systematic way after re-hydration with a droplet of saline by using a Leica phase contrast microscope at 400 times magnification (DC 1000, Warburg, Germany) according to the classification system published elsewhere [18]. In brief, lactobacillary grades (LBG) were classified and bacterial vaginosis (BV) flora was diagnosed on the basis of the typical granular microflora. Full BV was defined when the BV flora was observed all over the smear with presence of more than 20 % clue-cells or 'partial BV' if there were less than 20 % of clue cells with only areas with streaks of BV-like bacteria mixed with other morphotypes [19]. Aerobic vaginitis (AV) flora was defined and classified according to the presence of small bacilli exclusively or containing cocci in pairs or chains. LBGs, and presence of parabasal cells and leucocytes (see below) were added to the final AV score [20]. Evaluation of vulvovaginal candidosis (VVC) was made when unequivocal pseudohyphae and/or blastopores (‘snowman’ shape) were detected on the smears (Fig. 1a). In cases where the blastospores or hyphae were damaged or atypical, they were designated as 'possible Candida' (Fig. 1b). Leucocytes were quantified and evaluated if a granular or swallowed nuclei were present, determining their toxic form. Quantification of leukocytes was done by counting their number per high power field, and if more than ten (score 2 or more), by counting them as a ratio to the number of epithelial cells present: <10 per epithelial cell is scored as 2, more than 10 per epithelial cell as 3, and full field of leucocytes as 4. Presence of mucous cervicitis was considered if leucocytes were numerous and following a layer pattern. Evidence of cytolysis of epithelial cells was detected by the presence of cytoplasm remains and bare nuclei, with classification in slight or severe cases according to the extension on the smears.

Fisher's exact was used to determine statistical significance between groups. For normally distributed continuous variables Student's t-test was used.

## Results

Gynoflor® successfully decreased the vaginal pH, and improved the majority of signs and symptoms of vaginal atrophy as well as the vaginal maturity index (VMI), lactobacillary grades and the presence of moderate to severe AV [5]. These improvements mostly appeared immediately during initial therapy and improved further under maintenance therapy.

**Fig. 2:**
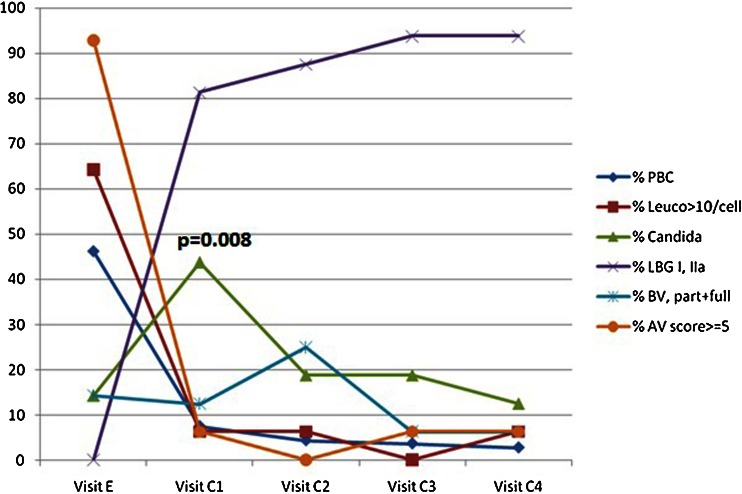
Change in flora characteristics of the vaginal microflora, inflammation and in quality of the vaginal epithelial cells at entry and during four follow up visits of 16 BC patients with aromatase inhibitor intake

**Fig. 3:**
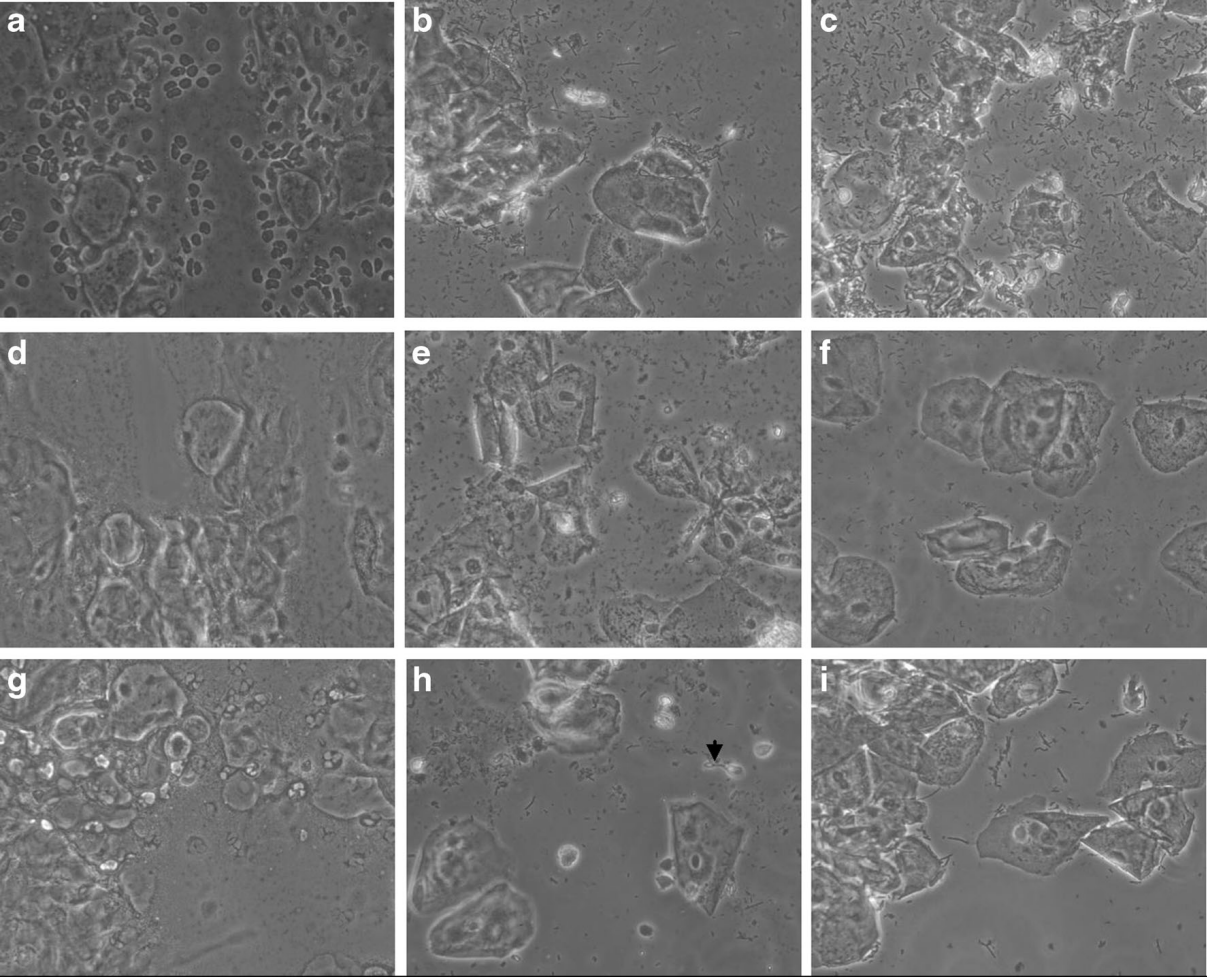
In patient 1, severe atrophy with lactobacillary grade (LBG) III, 50 % intermediate and 20 % parabasal epithelial cells are visualized at entry (**a**). The numerous red blood cells (RBC) indicated atrophic bleeding. After 1 week (**b**) and 12 weeks (**c**) of treatment the LBG improved dramatically to LBG I, no RBC, no inflammation and high numbers of superficial cells (above 80 %). In patient 2, lactobacillary grade (LBG) III, anaerobic microflora, 60 % intermediate and 20 % parabasal epithelial cells were seen at entry (**d**). After 1 week in patient 1, severe atrophy with lactobacillary grade (LBG) III, 50 % intermediate and 20 % parabasal epithelial cells are visualized at entry (**a**). The numerous red blood cells (RBC) indicated atrophic bleeding. After 1 week and 12 weeks of treatment the LBG has improved dramatically to LBG I, no RBC, no inflammation and high numbers of superficial cells (above 80 %). After 12 weeks of treatment the LBG has improved dramatically to LBG IIb and LBG I (**e** and **f**, respectively), and in both instances there was no inflammation and high numbers of superficial cells (> 80 % and > 90 %, respectively). In patient 3, severe atrophy was diagnosed with lactobacillary grade (LBG) III, 25 % intermediate and 60 % parabasal epithelial cells at initial visit (**g**). Treatment resulted in LBG IIA after one week and LBG I after 12 weeks of treatment, respectively, and above 80 % superficial cells in both instances. Remarkably in the smear taken after one week of treatment, a sperm cell was encountered (*arrow*, **h**)

At entry the majority of patients (81 %) had the most severe form of abnormal vaginal flora (LBG III), while the remaining had LBG IIb. Already after 2 weeks of initial therapy most patients presented with slightly disturbed (LBG IIa, 63 %) or normal (LBG I, 25 %) vaginal flora patterns, both considered normal, and no severely disturbed flora (LBG III) (Fig. 2). Further improvements were observed at the end of the maintenance therapy, at which point the majority of patients had normal (LBG I, 69 %) or slightly disturbed (LBG IIa, 25 %) vaginal flora. Changes in LBG from entry to all control visits as well as the further change observed during the maintenance therapy were statistically significant (*p* < 0.001) (Figs. 2 and 3).

Although there was a non-significant trend of diminishing BV flora towards the end of the study, the BV score showed no significant variations over time, and its presence was a rather rare event with two to four patients expressing BV flora at entry or control visits.

On the contrary, the AV score substantially improved during treatment. At entry most patients had moderate (31 %) or severe (50 %) AV, while after initial therapy most patients presented without (62.5 %) or with only light AV (37.5 %) flora (*p* < 0.01). After maintenance therapy no patient except for one (94 %) had AV. The improvements in AV score from entry to all respective control visits and further improvement have been observed after maintenance therapy and they were statistically significant. Remarkably, the inflammatory component, as assessed by the proportional number of leukocytes present, dropped dramatically from a score of 1.78 ± 0.70 to 1.06 ± 0.25 which was consistent till the end of the study. Heavy (>10 leukocytes/epithelial cell) and moderate leucocytosis (>10/high power field, but < 10/epithelial cell) were seen in two, respectively, in 7 of the 15 slides at entry (one slide unreadable) but only two had moderate and none severe leucocytosis at subsequent visits.

The presence of parabasal cells, a surrogate marker of vaginal maturity (also expressed by the VMI), and component of the AV score, dropped dramatically and continuously from a score of 3.4 ± 0.64 at entry to 2.0 ± 0.81 in V1, 1.6 ± 0.72 at V2, 1.5 ± 0.6 at V3 and 1.3 ± 0.60 at the final visit (*p*_trend_ < 0.01).

Finally, a surprising course of *Candida* presence became evident. Starting from a low rate of unequivocal *Candida* colonisation of 2/14 (14 %), a sudden rise to 7/16 (44 %) at C1 occurred, to return back to base levels of 19 %, 19 % and 13 % in the subsequent visits, respectively. This peak at C1 shows a significantly higher rate of unequivocal Candida than in the other visits (*p* = 0.035), and when the 'possible' Candida is taken into consideration, the difference is even more pronounced (10/16, 63 % at V1 vs 16/53, 25 %, *p* = 0.008). In four of seven women with Candida at C1, colonisation persisted at one (*n* = 1), two (*n* = 2) or all three subsequent visits (*n* = 1). Nevertheless, only one patient was symptomatic enough to take treatment, while the others did not require or receive any antimycotic treatment.

## Discussion

All estrogens are contra-indicated in hormone receptor positive BC women, however, it is necessary to reflect on safety of any possible treatment regimen for vaginal atrophy. The daily vaginal application of ultra-low dose 0.03 mg E3 in postmenopausal women results only in a small and transient increase in serum E3, but serum levels of E1, E2, FSH, LH and SHBG are not influenced leading to the conclusion this therapy could be safe even in hormone sensitive BC patients [5, 13, 21]. Furthermore, the proliferation of the vaginal epithelium during repeated daily therapy with 0.03 mg E3 and lactobacilli in BC women with vaginal atrophy reduces the absorption of E3 even further, in so far that after an initial therapy of 12 days the E3 serum levels did not increase at all in most of the women on maintenance therapy [5]. The typical side effects of estrogen (water retention, feeling of tension in the breasts, mastalgia, increase in weight, migraine headaches, visual disturbances, nausea, increase in blood pressure, bleeding from the uterus, etc.) nor endometrium proliferation were reported for Gynoflor® and ultra-low dose vaginal E3 in general [10, 13, 17, 21–25]. The Lactobacillus acidophilus strain contained in Gynoflor® is of human origin and has been shown in-vitro to possess the key beneficial properties for vaginal use. The strain produces lactic acid hydrogen peroxide, and bacteriocin and adheres well to epithelial cells, and thus significantly inhibits the growth and adherence of vaginal pathogens [16].

In the present study we could demonstrate that the improvement of the VMI coincides with the improvement of the vaginal bacterial microflora. This transition occurs immediately after start of therapy and is well maintained during maintenance therapy with three vaginal tablets weekly. At entry an increased number of parabasal cells (PBC) were present in almost all women, as markers of severe atrophy. Upon treatment, however, the mean number of PBC was strongly reduced, and the mean VMI was 70 % or more for the whole duration of the study. Reciprocally, LBG promptly improved to normal flora patterns in more than 80 % of women after initiation of therapy, and even to more than 90 % of women in the maintenance phases. Even though lactobacilli were absent (LBG III) in most patients at entry, BV was only present in 14 % of them, indicating most subjects with disrupted flora have AV rather than BV in this patient group. Remarkably, even with improving LBGs, the rate of BV hardly changed over time, and at control in two even a slight increase was seen. The latter can be related to resumed or increased sexual activity, as BV is known to be associated with the level of sexual intercourse. Unexpectedly, the Candida colonization rate rose three-fold immediately after initiation of therapy to 44 % of women, but declined to baseline rates after. Remarkably this decline occurred without treatment, as only one patient used antifungal medication. This observation seems to indicate that most of these findings are indicative of asymptomatic colonisation rather than symptomatic VVC. Still we would recommend to routinely check for VVC in patients treated for atrophic vaginitis, as the rate of Candida was higher than expected, especially in the first weeks after initiation of treatment with Gynoflor®. About the reason why Candida colonisation increased at first and then declined again, we can only speculate. It is known that a sudden change in flora, with improvement of the vaginal epithelium and LBGs due to treatment of BV, for instance, after treatment with metronidazole, can coincide with the occurrence of symptomatic VVC and that *Candida* is found more often in lactobacillus dominant flora. The subsequent decline after this initial peak, however, can possibly be ascribed to the protective effect of Gynoflor®, as its *L. acidophilus* is known for its beneficial properties against pathogens [16].

Finally, the treatment with low E3 combined with lactobacilli had a profound and unexpected effect on vaginal inflammation. Probably this reduction of immune competent cells and leukocytes is a favourable finding not only for symptomatic women like BC patients or other menopausal women with vaginal atrophy, but could also turn out to be beneficial for pregnant women with disrupted flora and at risk of preterm delivery. Studies testing the potential benefits of such probiotic products in pregnancy are urgently needed.

Of course, we realize that our conclusions are based on a small number of patients and need to be confirmed in larger series. However, as the data were retrieved from a phase 1 safety trial in breast cancer patients, higher numbers were not feasible in this setting. Also as a compensation for the small numbers, microscopic studies were done in great detail (according to the Femicare protocol [18]), allowing minimal changes to be detected more readily. Furthermore, the findings were so dramatic and universal that even in low numbers they were clear and outstanding. That larger numbers would significantly change the conclusions therefore seems unlikely. One could argue that cultures and/or molecular microbioma research could have brought a broader insight in the changes brought along in the vaginal microflora. However, positive cultures or PCR findings would still not reflect the health status of the flora as good as microscopy does [26]. An exception could be the increased detection rate of *Candida* organisms by use of extra techniques, but in that case most likely only representing asymptomatic colonisation would be detected.
